# Chromosomal Location of *Pm12*—A Novel Powdery Mildew Resistance Gene from *Avena sterilis*

**DOI:** 10.3390/genes13122409

**Published:** 2022-12-19

**Authors:** Tomasz Ociepa, Sylwia Okoń

**Affiliations:** Institute of Plant Genetics, Breeding and Biotechnology, University of Life Sciences in Lublin, Akademicka 15, 20-950 Lublin, Poland

**Keywords:** *Avena*, DArTseq, genetic mapping, powdery mildew, resistance gene

## Abstract

Identification of new, effective disease resistance genes is a very important aspect of plant breeding. Also important is the precise location of individual loci and tagging them with DNA markers for marker assisted selection. The aim of the present study was identification of the molecular markers linked with *Pm12*, a new effective resistance gene to powdery mildew, and their location in the oat genome. The analysis was performed on 167 F_2_ individuals from a hybrid of Fuchs × CN67383, with the status of the locus in each individual verified by progeny test in F_3_. Segregation ratios confirmed the monogenic nature of resistance. Making use of the sequence data of DNA markers and the oat OT3098 v2 genome reference assembly, *Pm12* is located on chromosome 7C. A comparison was also made with the reference consensus map, to which there are more reports of mapped genes to date. The mapping results suggest that *Pm12* is located in the interval 103.8–111.7 cM on this map. No powdery mildew resistance locus has been identified in this region so far, suggesting that *Avena sterilis* CN67383 carries a novel locus offering effective resistance in oat breeding. The information included in the oat genome annotation allowed for the identification of candidate genes in the close region of the marker cluster for *Pm12*. This information may provide an interesting source of further analysis of the pathways of various genes in response to the stress of powdery mildew infection.

## 1. Introduction

Common oat (*Avena sativa* L.) is one of the most economically important cereals next to maize, rice, wheat, barley, sorghum and millet. It is grown all over the world, but most of its production is concentrated in North America, Europe and Asia. The main producers of oat are Russia, Canada, the United States, Finland and Poland [1].

Like other cereals, oat is vulnerable to pathogens that cause quantitative crop losses. Climate changes in recent years have resulted in the spread of fungal diseases. Therefore, it is necessary to increase the resistance of oat to counteract fungal pathogens [2,3]. Powdery mildew caused by *Blumeria graminis* DC. f. sp. *avenae* Em. Marchal is one the most important foliar diseases of oat [4]. Crops in cold and humid regions, where rain occurs in the initial stage of plant vegetation and temperatures in this period are relatively low are the most vulnerable to disease development. Oat grain yield losses caused by powdery mildew amount to 5–10%, and in years favorable for infection they can be as high as 39% [5]. This has been reported as a serious problem in the UK [6], north-western and central Europe [7] and the USA [8]. Moreover, the disease has spread in recent years to areas where it had not occurred previously, such as China [3] and the north-western Himalayas [2].

Based on the literature, 11 genes have been identified that confer resistance to powdery mildew in oat [9,10,11]. Research carried out in recent years has shown that the *Pm2*, *Pm4*, *Pm5* and *Pm7* genes are the most effective against this pathogen [12,13]. A small group of effective genes need to introduce more sources of resistance that would ensure sustained cultivation of healthy plants under various environmental conditions.

Wild relatives of cultivated oat are an excellent source of resistance genes. *A. sterilis* is one of the hexaploid wild species often used in oat breeding programs as a source of valuable genes. Okoń et al. [14,15] found that *A. sterilis* is also a valuable source of genes against *B. graminis* f. sp. *avenae*. Genotype CN67383 has been characterized as an effective source of resistance to powdery mildew. It ensured a high level of resistance in physiological tests based initially on 13 and then on 50 *B. graminis* f. sp. *avenae* isolates with different levels of virulence [16]. On the basis of the host–pathogen tests, the authors confirmed that this genotype had a different infection profile than the lines and cultivars with previously described powdery mildew resistance genes, suggesting that it carried a new resistance locus. The present study is the next stage of research on a new, effective resistance gene identified in the *A. sterilis* genotype CN67383.

The aim of this study was to determine the inheritance of this new, effective resistant gene to powdery mildew in oat, named *Pm12*, and the chromosomal location of markers closely linked to this gene using the DArTseq method. In addition, we would like to confirm or exclude the possibility that it could be a different gene than those previously described for powdery mildew resistance.

## 2. Materials and Methods

### 2.1. Mapping Population

The subject of the study was an F_2_ population derived from the cross between the susceptible cultivar Fuchs and *A. sterilis* genotype CN67383. This genotype has been identified as a valuable source of resistance against oat powdery mildew [15] and characterized as highly effective against oat powdery mildew pathotypes present in Poland in 2010–2017 [16]. One hundred and sixty-seven individuals from the F_2_ population were phenotyped based on the host–pathogen test, as explained below. After tests, all individuals were planted in the experimental plot. F_3_ generation seeds were collected from each F_2_ individual. Fifteen seeds from each F_3_ plant were also tested using host–pathogen methodology.

### 2.2. Phenotyping—Resistance Tests

In order to determine the resistance of individuals in F_2_ and F_3_ populations, 167 individuals were tested using two *B. graminis* f. sp. *avenae* isolates with different geographic origin. The isolate marked with the symbol CZE came from eastern Poland, while the CHR isolate was derived from western part of the country. Moreover, the isolates differed in the level of virulence in relation to the described powdery mildew resistance genes. According to the nomenclature proposed by Okoń et al. [17], the CZE isolate was classified as pathotype TBBB, which means that it was virulent to *Pm1*, *Pm3*, *Pm6* and *Pm3+8* genes. The CHR isolate was virulent to *Pm1*, *Pm3*, *Pm6*, *Pm3+8*, *Pm9* and *Pm11* and represented pathotype TBHB. As a susceptible control, the leaves of the cultivar Fuchs were used. Host–pathogen tests were carried out on the first leaves of 10-day-old seedlings. Leaf segments of each individual were placed in 12-well culture plates with 6 g/L agar and 35 mg/L benzimidazole. The plates with the leaf segments were inoculated in a settling tower by spreading 500–700 powdery mildew spores per cm^2^. The plates were then incubated in a growing chamber at 17 °C and an illuminance of approximately 4 kLx. Reaction types on each individual of the F_2_ and F_3_ populations were determined 10 days after inoculation and scored according to a 0–4 modified scale [18], where 0 = no infection, no visible symptoms; 1 = highly resistant, fungal development limited, no sporulation; 2 = moderately resistant, moderate mycelium with some sporulation; 3 = moderately susceptible, extensive mycelium, more sporulation; 4 = highly susceptible, large colonies and abundant sporulation. If disease symptoms were scored as 0, 1 or 2, the individuals were classified as resistant. If disease symptoms were scored as 3 or 4, the individuals were classified as susceptible. Plants segregated into resistant and susceptible types in both tests. The segregation ratio of F_2_ and F_3_ populations was analyzed using Chi-square tests for goodness of fit.

### 2.3. Genotyping and Linkage Map Construction

Ninety-two randomly chosen individuals from the F_2_ population and parental forms were used for genotyping based on the DArTseq method, using Illumina next-generation sequencing. The DArTseq analysis was performed at Diversity Arrays Technology Pty Ltd. (Canberra, Australia) according to the company’s methodology. Genomic DNA was extracted from fresh 10-day-old leaves using DNeasy Plant Mini Kit (Qiagen, Hilden, Germany). DNA integrity and quality were evaluated by electrophoresis on 1.5% agarose gel. DNA concentration was determined with NanoDrop2000 spectrophotometry and normalized to 50 ng μL^−1^. 

Using DArTsoft, the silicoDArT markers were scored as binary data (0 = absence or 1 = presence), and the SNP markers were scored as data in the 0–2 scale (where 0 is for homozygous reference state, 1 for heterozygous, and 2 for homozygous alternate state). Several quality parameters, such as call rate, one ratio, polymorphism information content (PIC), avgReadDepth, StDevReadDepth, qpmr and reproducibility, were also calculated.

In order to better compare the results to the consensus map, a genetic map was constructed from the obtained results using the software IciMapping version 4.1 [19]. The obtained silicoDArT and SNP markers were filtered according to the following parameters: not more than 5% of missing rate and not lower than 0.05 of distortion p value. The Haldane mapping function was used to convert recombination fraction into map distance. Logarithm of the odds ratio (LOD) score ≥3.0, nnTwoOpt algorithm and the sum of adjacent recombination frequency (SARF) were used for map grouping, ordering and rippling, respectively.

### 2.4. QTL Analysis

QTL analysis to detect main-effect QTL was conducted by using QTL IciMapping version 4.1 [19], using functions (BIP) to map biparental populations, following the Inclusive Composite Interval Mapping of Additive and Dominant (ICIM-ADD) and Single Marker Analysis (SMA) mapping methods within the software. In order to select only the narrow region with the highest likelihood ratio statistic, an LOD score above 10 was considered as suggestive of a QTL. Additive QTL was detected using a 1.0 cM step in scanning.

### 2.5. Chromosomal Localization and Gene Annotation Matching

The markers selected in the QTL analysis were analyzed for their localization on the oat chromosomes. Twenty-one SNPs and one silicoDArT marker sequence were used to perform local BLASTn (https://wheat.pw.usda.gov/blast, accessed on 1 June 2022) analysis on the PepsiCo OT3098 Hexaploid Oat Genome ver. 2 with the gene annotation (https://wheat.pw.usda.gov/jb?data=/ggds/oat-ot3098v2-pepsico, accessed on 1 June 2022). To test the hypothesis that the *Pm12* gene markers map in a different region from the other defined mildew resistance genes, all markers in the DArTseq genotyping were assigned to their counterparts in the consensus map described by Bekele et al. [20], available in T3/Oat database (https://triticeaetoolbox.org/oat/viroblast/viroblast.php, accessed on 1 June 2022), and their connections with the created Fuchs × CN67383 genetic map were visualized using Circos software [21]. In both cases, filters with the lowest “e-value” = 1 × 10^−5^ and the greatest identity ≥95% were used. With the use of the JBrowse genomic viewer and filters enabling the display of information about the annotated genes, the analysis of the matching of marker sequences to given genes in the genome was performed.

## 3. Results

One hundred and sixty-seven individuals from the F_2_ Fuchs × CN67383 population were assayed in two independent host–pathogen tests using different *B. graminis* f. sp. *avenae* isolates. In both tests, segregation for resistant and susceptible plants was obtained. The numbers of resistant and susceptible individuals were very similar: 127 and 126 resistant and 40 and 41 susceptible individuals were identified in the tests based on the CHR and CZE isolates, respectively. Chi-square tests for goodness of fit in both cases did not deviate from expectations under the model of 3 resistant: 1 susceptible (Table 1). Host–pathogen tests were also performed in individuals from the F_3_ population to confirm monogenic inheritance of the trait. In the F_3_ generation, 15 individuals representing each plant of the F_2_ generation were tested. Tests in the F_3_ generation were also carried out on the basis of the same *B. graminis* f. sp. *avenae* isolates. The obtained segregation was close to the ratio 1 resistant: 2 segregating: 1 susceptible, thereby confirming the monogenic inheritance of resistance conditioned by the *Pm12* gene (Table 1).

DArTseq analysis of the F_2_ mapping population Fuchs × *A. sterilis* CN67383 was performed to identify markers associated with the *Pm12* resistance gene. Ninety-two randomly selected individuals were genotyped, and a total of 33,702 silicoDArT and 15,115 SNPs were identified. Markers with a percentage of missing data greater than 5% and a p-value less than 0.05 were deleted. Among them, 416 silicoDArT and 3577 SNP markers were polymorphic within the analyzed population.

**Figure 1:**
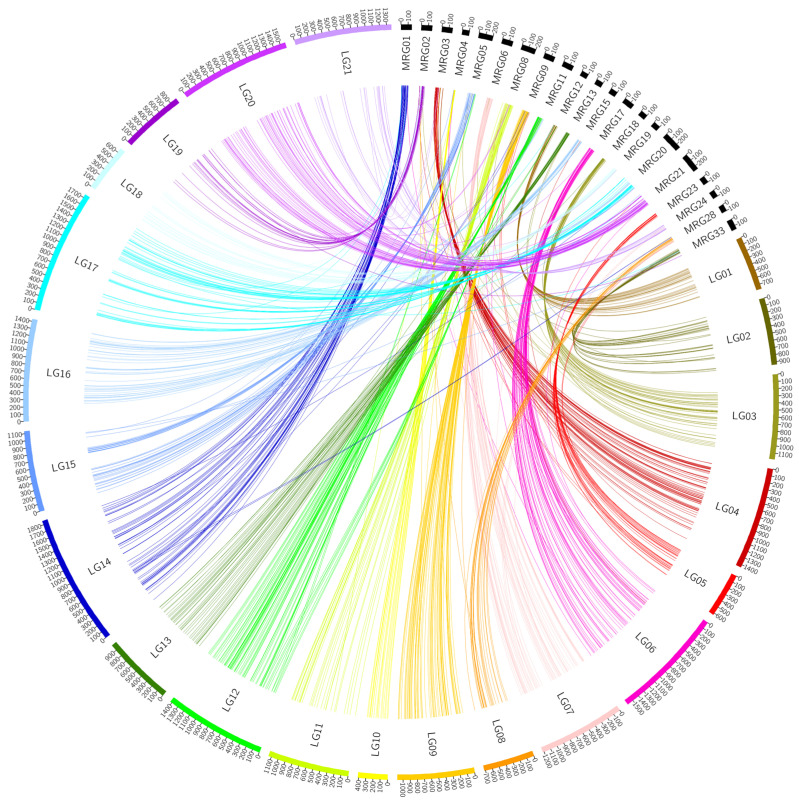
A graphical representation of the Fuchs × CN67383 genetic map and their connection to the groups of the Bekele et al. consensus map.

After discarding redundant markers, 3577 SNP and 416 silicoDArT markers were used to construct the genetic map (Figure 1) and for further QTL analysis. Detailed information about the constructed Fuchs × CN67383 genetic map is presented in Table 2. Each of the obtained 21 LG groups from the constructed map was associated with a unique Mrg group of the consensus map [20]. This was confirmed by BLASTn analysis of the markers derived from both maps. The LG14 group had the most markers and the greatest length (1853.45 cM). The total genetic distance of the linkage map was 24,209.96 cM.

**Figure 2:**
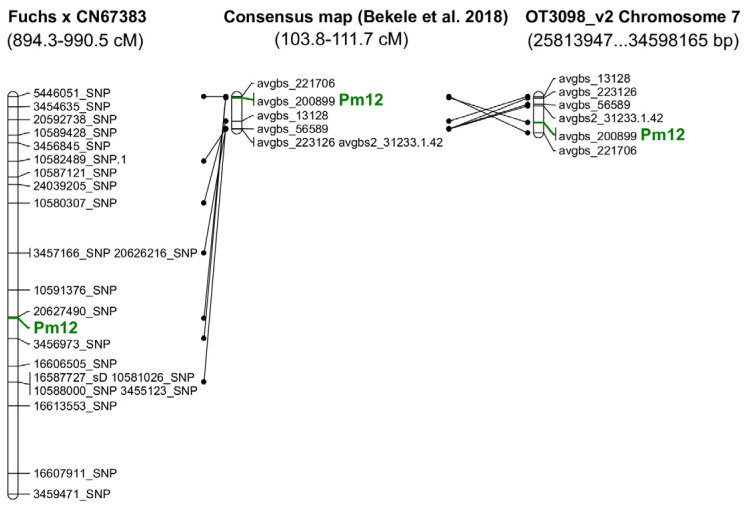
Location of the gene and markers linked to *Pm12* on genetic maps and on chromosome 7 of the reference genome [20].

QTL analysis using the SMA algorithm indicated 22 markers with LOD higher than 10 (Supplementary Material Table S1). QTL analysis using the ICIM-ADD algorithm shows three regions with LOD higher than 10 (Supplementary Material Table S2). All of the markers (21 SNPs and one silicoDArT) selected in both analyses were located on LG9 of the Fuchs × CN67383 map. Based on T3oat database annotations, some of the acquired silicoDArT and SNP markers could be located on the previous oat consensus map [20]. The highest number of markers was associated with the Mrg09 linkage group. The markers were separated by genetic distance ranging from 103.8 to 111.7 cM (Supplementary Material Table S1, Figure 2).

For genome OT3098 v2, all SNP and silicoDArT markers selected in the QTL analysis were located on chromosome 7C. In both cases, filters with the lowest “e-value” = 1 × 10^−5^ and the greatest identity ≥95% were used.

Based on the information available in the annotation for the OT3098 v2 genome, the analysis of the matching of the sequences of SNP and silicoDArT markers to the genes given in the annotation was performed. Six marker sequences were mapped within specific genes whose genes ontology shows that they participate in molecular processes such as ATP binding, protein kinase activity, cysteine-type endopeptidase activity, thiol-dependent deubiquitinase and aspartate kinase activity (Supplementary Material Table S3).

## 4. Discussion

The search for new sources of resistance is a very important element in protecting plants against pathogens. Detailed characteristics of the inheritance method of new genes, as well as their chromosomal localization and selection of markers linked to the analyzed gene, are important tools in plant breeding.

In the presented study, based on the host–pathogen tests, we showed that resistance to powdery mildew, previously identified by Okoń et al. [15] in the *A. sterilis* genotype CN67383, is conditioned by a single dominant gene. This has been proved by segregation analyses in both the F_2_ and F_3_ generations. Specifying the inheritance of resistance source allows for its better use in increasing the resistance of cultivated forms.

The host–pathogen test in Okoń and Ociepa [16], performed on the basis of 50 differentiated isolates of *B. graminis* f. sp. *Avenae,* showed that the infection profile of the CN67383 genotype differs from the infection profile of previously described genes for resistance to powdery mildew. These results suggested that the resistance to powdery mildew identified in the CN67383 genotype is conditioned by a new resistance gene that has not been described so far. To confirm this hypothesis, we made an attempt to localize the *Pm12* gene in the oat genome. The mapping population was genotyped using the DArTseq method. This method reduces the complexity of the genome by digestion with restriction enzymes followed by sequencing of short reads. The choice of a combination of restriction enzymes allows for the isolation of highly informative low-copy fragments of the genome. DArTseq analysis generates two datasets. The first contains dominant markers (silicoDArT); the second includes codominant markers with marked single nucleotide polymorphisms (SNP). This method produces at least three times more dominant markers than the conventional DArT method [22]. Additional advantages of DArTseq technology are its suitability for species such as barley [23], oat [10], rye [24] or wheat [25] and its high popularity among crops with the non-sequenced genomes, for example, pea [26]. Therefore, this method was used in the discussed experiment because the oat genome was developed relatively recently and most of the work on the *Pm* genome mapping was based on the consensus map [20] or older versions.

The results of BLASTn analysis showed that all silicoDArT and SNP markers correlated with Pm12 were located on chromosome 7C. With reference to the consensus map of Bekele et al. [20], this gene was most likely located on the Mrg09 linkage group at a genetic distance of 103.8–111.7cM. Thus far, only a few powdery mildew resistance genes have been identified and mapped. Herrmann and Mohler [9] mapped *Pm9* to Mrg21 and *Pm10* to Mrg03. They have been mapped on chromosome 4D for *Pm9* and chromosome 5C for *Pm10*. Ociepa et al. [10] located five markers closely linked with the *Pm11* resistance gene on Mrg12 at genetic distance 14.1–17.0 cM. These markers in the PepsiCo OT3098 v2 are located on chromosome 7A. Mohler [27], based on SNP data and AFLP and RFLP markers from a previous study [28], mapped the *Pm3* gene to group Mrg18, which was determined to represent chromosome 1A. According to the author, *Pm3* was located at 67.7–72.6 cM in Mrg18 of the consensus map of Bekele et al. [20]. For the remaining powdery mildew resistance genes, the literature does not provide exact chromosomal location linkage groups. However, Herrmann and Mohler [9] summarized the resistance gene data and indicated that the *Pm1* gene was probably located in Mrg11 or Mrg05 when referencing the map published by Chaffin et al. [29]. The *Pm6* gene is likely located in the Mrg03 or Mrg15 groups. At present, these genes are no longer effective against the existing *B. graminis* f. sp. *avenae* isolates, and their importance in breeding programs is decreasing [12]. The *Pm4* gene is most likely located in Mrg04, while the *Pm5* gene is most likely located in Mrg20 [11,30]. The *Pm7* gene has been assigned to the Mrg12 group. The location of the *Pm2* gene is unknown and has not been introduced into cultivated forms, and despite its high efficiency, it is not used in breeding programs [31,32].

Taking into account the unique infection profile characterized by Okoń and Ociepa [16] in the host–pathogen tests and the location of the *Pm12* gene on chromosome 7C, we can confidently state that the resistance identified in the *A. sterilis* CN67383 genotype is conditioned by a new, hitherto undescribed gene.

Okoń et al. [17] and Cieplak et al. [33] showed that the resistance of the *Pm12* gene is maintained at a level that allows for a satisfactory protection of plants against *B. graminis* f. sp. *avenae*. The frequency of virulence in relation to this gene remained at the level of about 20% in the following years of research. However, this gene was highly effective against isolates collected in Poland, Germany and Ireland. Pathogen isolates from Finland and the Czech Republic broke the resistance of this gene. Therefore, it is necessary to conduct constant research on the effectiveness of resistance genes in various regions of the world so that it is possible to select the most effective sources of resistance.

The development of next-generation sequencing technology allowed for oat genetic maps with increasing coverage of molecular markers [20,29,34]. In 2020, PepsiCo and Corteva Agriscience announced the DNA assembly of 21 chromosomes of a North American oat variety (OT3098) using the PacBio long-read technology, which was simultaneously made publicly available via the GrainGenes website. Work on more accurate versions of this genome is ongoing. The proof is the release of the second version with the gene annotations. Adding an annotation to the developed genome allows selecting genes that may be related to the plant’s defence response to an attack by a specific pathogen [35]. In the case of *Pm12*-related markers, six were selected, the sequences of which were 100% matched within the sequence of the genes that are involved in biological processes such as protein autophosphorylation, cell wall organization, defence response, ubiquitin-dependent catabolic process of proteins and biosynthesis of amino acids such as homoserine, lysine or threonine. Research on the expression of similar genes was carried out on other plant models during biotic or abiotic stress. For example, IQD5 genes play a crucial role in responses to drought stress in Chinese cabbage [36]. Studies on rice show that the EXPA7 gene is regulated under cold stress conditions, possibly facilitating shoot elongation [37]. Zhang et al. [38] performed a comparative phosphoproteome analysis of the developing grains in bread wheat (*Triticum aestivum* L.) under well-watered and water-deficit conditions. These studies confirmed that the process ubiquitination mediates diverse cellular processes, especially with respect to proteasome composition, ribosome assembly/translation, carbohydrate metabolism, signal transduction and photosynthesis.

Recent advances in genomic resources mean that genomic selection is now more readily available for use in oat breeding programs. Genomic selection (GS) is a form of marker-assisted selection (MAS) that utilizes a very large number of genetic markers covering the whole genome. In this case, all quantitative trait loci (QTL) are closely linked on chromosomes with at least one marker. The exact location of individual resistance genes provides important information in the context of modern plant breeding.

## 5. Conclusions

The conducted physiological tests confirmed that the resistance identified in our previous studies on *A*. *sterilis* CN67383 is conditioned by a single dominant gene. We localized the new *Pm12* gene on the 7C chromosome, confirming that it is a new, hitherto undescribed gene for resistance to *B. graminis* f. sp. *avenae*. The high efficiency of this gene makes it possible to use it in breeding programs as a single resistance gene. However, a better solution would be to build gene pyramids that could provide long-term resistance to different races of the pathogen.

## Figures and Tables

**Table 1:** Seedling response and segregation ratio of F_2_ and F_3_ families derived from Fuchs ×CN67383 cross-inoculated with different *B. graminis* DC. f. sp. *avenae* Em. Marschal isolates.

F_2_ Population (Fuchs × CN67383)					F_3_ Population (Fuchs × CN67383)				
Powdery Mildew Isolates	Resistant	Susceptible	χ^2^ 3:1	*p*-Value	Resistant	Segregating	Susceptible	χ^2^ 1:2:1	*p*-Value
CHR	127	40	0.127	0.721	42	85	40	0.143	0.930
CZE	126	41	0.032	0.858	39	87	41	0.431	0.806

**Table 2:** Number of markers and genetic distance for each linkage group of the Fuchs × CN67383 map with matching to the merged groups of the Bekele et al. map [20].

Linkage Groups of Fuchs × CN67383 Genetic Map	Matching to Merged Groups of Bekele et al. Consensus Map	Number of Markers in Each Linkage Group of Fuchs × CN67383 Genetic Map	Length [cM]
LG1	Mrg12	111	765.2
LG2	Mrg33	135	939.07
LG3	Mrg18	197	1188.23
LG4	Mrg03	222	1438.25
LG5	Mrg23	122	623.59
LG6	Mrg17	266	1590.88
LG7	Mrg06	214	1216.61
LG8	Mrg28	142	717.75
LG9	Mrg09	231	1081.67
LG10	Mrg04	90	412.37
LG11	Mrg08	178	1141.89
LG12	Mrg11	249	1425.05
LG13	Mrg13	226	988.75
LG14	Mrg01	290	1853.45
LG15	Mrg05	165	1138.56
LG16	Mrg15	225	1434.57
LG17	Mrg20	224	1752.05
LG18	Mrg19	122	668.99
LG19	Mrg02	122	899.02
LG20	Mrg21	259	1568.67
LG21	Mrg24	199	1365.34
TOTAL		3989	24,209.96

## Data Availability

Data, plant materials and *B. graminis* f. sp. *avenae* isolates are available upon request from the corresponding authors.

## Supplementary Materials

The following supporting information can be downloaded at https://www.mdpi.com/article/10.3390/genes13122409/s1. Supplementary material Table S1: Chromosomal localization and genetic distance of obtained silicoDArT and SNP markers; Supplementary material Table S2: Regions with LOD higher than 3.5 in QTL analysis; Supplementary material Table S3: Gene candidates.
